# Effects of occlusal disharmony on susceptibility to atrial fibrillation in mice

**DOI:** 10.1038/s41598-020-70791-8

**Published:** 2020-08-13

**Authors:** Kenji Suita, Yuka Yagisawa, Yoshiki Ohnuki, Daisuke Umeki, Megumi Nariyama, Aiko Ito, Yoshio Hayakawa, Ichiro Matsuo, Yasumasa Mototani, Yasutake Saeki, Satoshi Okumura

**Affiliations:** 1 Department of Physiology, Tsurumi University School of Dental Medicine, 2-1-3 Tsurumi, Tsurumi-ku, Yokohama, 230-8501 Japan; 2 Department of Orthodontics, Tsurumi University School of Dental Medicine, Yokohama, 230-8501 Japan; 3 Department of Pediatric Dentistry, Tsurumi University School of Dental Medicine, Yokohama, 230-8501 Japan; 4 Department of Dental Anesthesiology, Tsurumi University School of Dental Medicine, Yokohama, 230-8501 Japan; 5 Department of Periodontology, Tsurumi University School of Dental Medicine, Yokohama, 230-8501 Japan

**Keywords:** Physiology, Cardiology, Diseases, Medical research, Molecular medicine, Pathogenesis, Risk factors

## Abstract

Tooth loss or incorrect positioning causes occlusal disharmony. Furthermore, tooth loss and atrial fibrillation (AF) are both risk factors for ischemic stroke and coronary heart disease. Therefore, we hypothesized that occlusal disharmony-induced stress increases susceptibility to AF, and we designed the present study to test this idea in mice. Bite-opening (BO) was done by cementing a suitable appliance onto the mandibular incisor to cause occlusal disharmony by increasing the vertical height of occlusion by 0.7 mm for a period of 2 weeks. AF susceptibility, evaluated in terms of the duration of AF induced by transesophageal burst pacing, was significantly increased concomitantly with atrial remodeling, including fibrosis, myocyte apoptosis and oxidative DNA damage, in BO mice. The BO-induced atrial remodeling was associated with increased calmodulin kinase II-mediated ryanodine receptor 2 phosphorylation on serine 2814, as well as inhibition of Akt phosphorylation. However, co-treatment with propranolol, a non-selective β-blocker, ameliorated these changes in BO mice. These data suggest that improvement of occlusal disharmony by means of orthodontic treatment might be helpful in the treatment or prevention of AF.

## Introduction

Atrial fibrillation (AF), one of the most prevalent arrhythmias, is an independent risk factors for strokes and heart failure, and is associated with increased mortality and morbidity^1^. Established risk factors for AF include high blood pressure, elevated body mass index, diabetes, and cigarette smoking^2^, but these appear to account for only just over a half of the total incidence of AF. Recent studies indicate that chronic stress is also a potential risk factor for AF via mechanisms involving increased inflammation and increased activity of the autonomic nervous system, hypothalamus–pituitary–adrenal axis, and renin–angiotensin–aldosterone system^3–5^.

A significant association between poor oral health and cardiovascular disease was first reported in 1989^6^. Since then, extensive studies have shown that periodontal disease is associated with elevations of several markers of chronic inflammation, and it might also be a factor in the etiology of cardiovascular disease^7–11^. However, men with 0–10 teeth was demonstrated to be a significantly higher risk of cardiovascular disease than men with 25–32 teeth, independent of the history of periodontal disease^12^, suggesting that other factors, such as occlusal disharmony, might contribute to the association between oral health and cardiovascular disease.

Occlusal disharmony has been shown to elevate the plasma level of corticosteroid, a marker of chronic stress, resulting in stimulation of the hypothalamus–pituitary–adrenal axis^13^. More recently, occlusal disharmony was suggested to cause systemic diseases, such as cognitive dysfunction and osteoporosis^14,15^.

Stress can influence the level of circulating catecholamine, which stimulates ß-adrenergic receptor (ß-AR) and triggers diverse intracellular pathways^16^. The chronic secretion and circulation of catecholamine to produce physiological responses when they are not required may result in pathological consequences in cardiac tissue, drastically affecting cardiac function ^17,18^. The stimulation of ß-ARs activates adenylyl cyclase (AC) by coupling with Gsα. AC in turn converts adenosine triphosphate (ATP) into cyclic adenosine monophosphate (cAMP), which facilitates downstream signaling via protein kinase A/exchange protein activated by the cAMP (Epac) signaling pathway^19–22^. However, the effects of occlusal disharmony on the downstream ß-AR/Gsα/cAMP signaling remain poorly understood.

**Figure 1:**
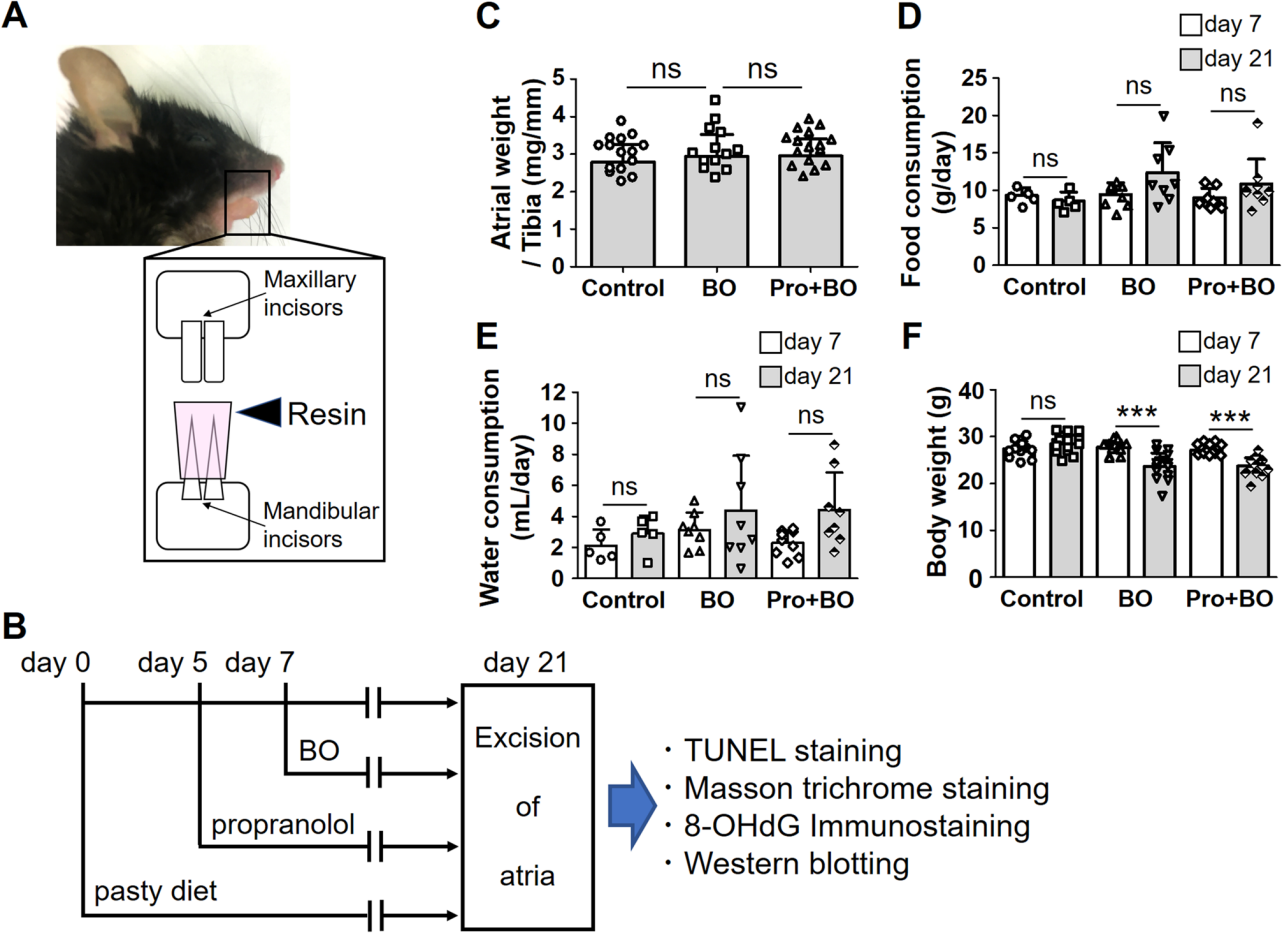
Schematic illustration of bite-opening, experimental procedure, atrial weight, daily consumptions of food and water. (**A**) Schematic representation of a bite-opening (BO) in the form of a 0.7 mm increase in the vertical height of occlusion, obtained by cementing a composite resin onto the mandibular incisors to cause occlusal disharmony in mice. (**B**) The standard pellet food was changed to paste food at 7 days before the BO treatment (day 0) with or without propranolol, which was administered 2 days before BO treatment (day 5) via drinking water (80 mg/kg/day). (**C**) No significant difference in atrial weight in terms of atrial weight per tibia length ratio (mg/mm) at day 21 among the Control (*n* = 15), BO (*n* = 13) and Pro + BO (*n* = 16) groups (*P* = NS vs. Control by one-way ANOVA). (**D**, **E**) No significant difference in daily consumption of food (**D**) or water (**E**) was observed at day 21 (gray bar), compared to that at day 7 (white bar), among the Control (*n* = 5), BO (*n* = 8) and Pro + BO (food consumption: *n* = 9, water consumption: *n* = 8) groups (*P* = NS vs. day 21 by two-way ANOVA). Data show means ± SD and scattered dots show individual data. (**F**) No significant difference in body weight (g) at day 21 (gray bar), compared to that at day 7 (white bar), among the Control (*n* = 16), BO (*n* = 15) and Pro + BO (*n* = 17) groups (*P* = NS vs. at day 21 by two-way ANOVA).

Therefore, the aim of this study is to clarify the relationship between occlusal disharmony and AF using a BO mouse model (Fig. 1A, B)^13,15,23–28^.

## Results

### Effects of BO on atrial weight and consumption of food and drinking water

We examined the atrial weight in terms of atrial weight per tibial length ratio and found that all three groups showed similar values (control group (Control) (*n* = 15); 2.8 ± 0.5 mg/mm, BO (*n* = 13); 2.9 ± 0.6 mg/mm, Pro + BO (*n* = 16); 3.0 ± 0.5 mg/mm, P = NS for BO and BO plus propranolol group (Pro + BO) vs. Control) (Fig. 1C).

We monitored the daily consumption of food and water per mouse during the experimental period. Consumption of food (Fig. 1D) before (day 7) and after (day 21) the BO treatment did not show significant difference among the three groups (day 7 vs. day 21; Control (*n* = 5): 9.3 ± 1.0 vs. 8.6 ± 1.2 g/day, BO (*n* = 8): 9.3 ± 1.6 g vs. 12.3 ± 4.0 g/day, Pro + BO (*n* = 9): 9.0 ± 1.2 g vs. 10.8 ± 3.3 g/day, *P* = not significant (NS) vs. day 21, in each case).

Consumption of water (Fig. 1E) before (day 7) and after (day 21) the BO treatment also did not show any significant difference among the three groups (day 7 vs. day 21; Control (*n* = 5): 2.1 ± 1.0 vs. 2.9 ± 1.2 mL/day, BO (*n* = 8): 3.1 ± 1.1 vs. 4.4 ± 3.7 mL/day, Pro + BO (*n* = 8): 2.3 ± 0.8 vs. 4.4 ± 2.4 mL/day, *P* = NS vs. day 21, in each case).

### Effects of BO on body weight

Body weight (BW) in the Control group showed no significant change during the experimental period. Conversely, BW gradually decreased in the BO and Pro + BO groups and reached a minimum at 4 days after the BO treatment (day 11), in accordance with previous findings^15,24^. After that, the BW of the BO and Pro + BO groups gradually increased, but did not reach the preoperative level during the experimental period (day 7 vs. day 21; Control (*n* = 16); 27.4 ± 1.6 g vs. 28.5 ± 1.9 g, BO (*n* = 15); 27.7 ± 1.5 vs. 23.6 ± 2.9 g and BO + Pro (*n* = 17); 27.1 ± 1.1 vs. 23.7 ± 1.8 g, ***P < 0.001 vs. day 7, in each case) (Fig. 1F).

### Effects of BO on electrophysiological parameters and susceptibility to AF

**Figure 2:**
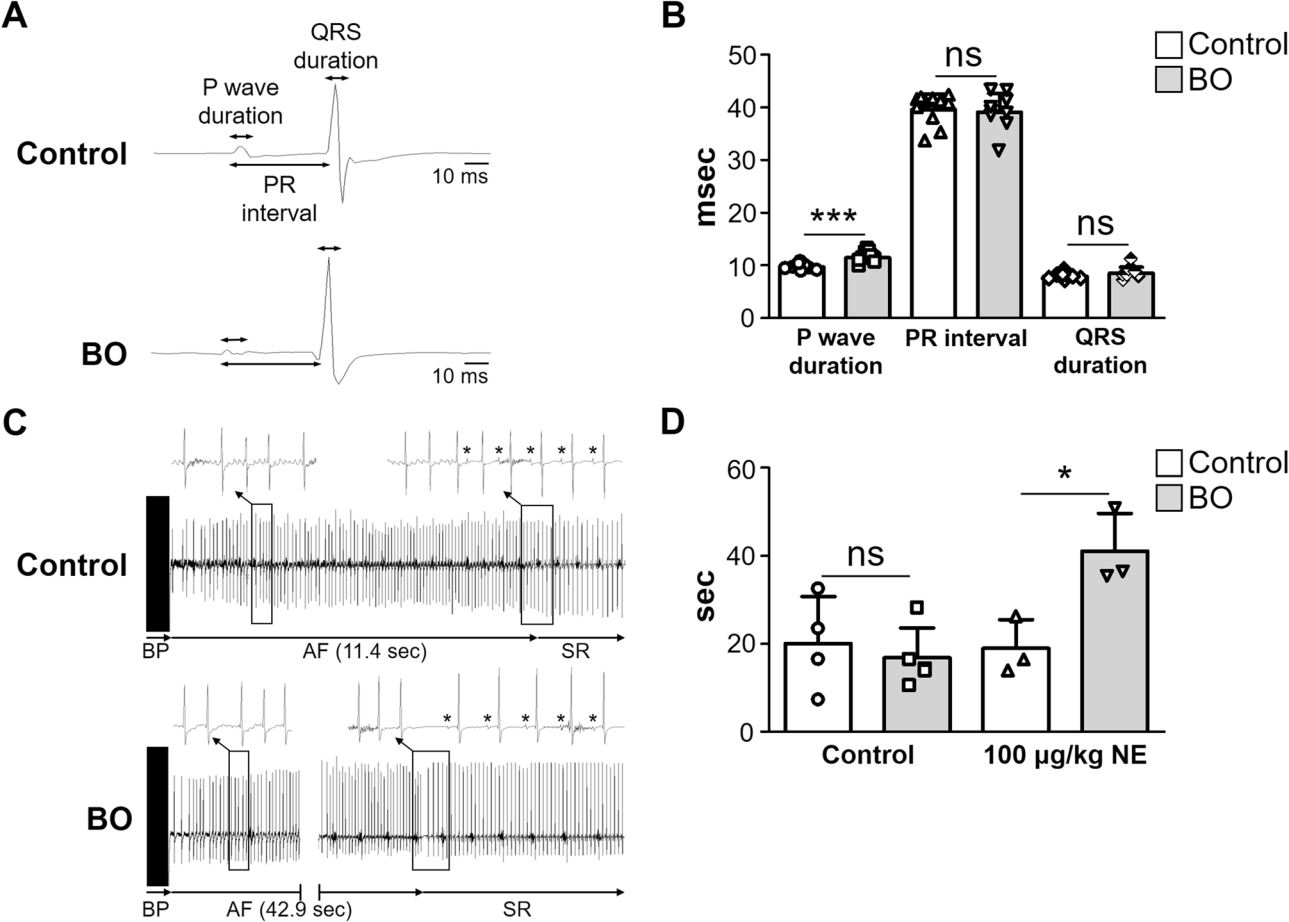
Effects of BO on electrophysiological parameters and susceptibility to AF. (**A**) Representative traces of surface ECG in Control (*upper panel*) and BO (*lower panel*) mice, respectively. P wave duration, PR interval, and QRS duration are indicated by arrows. (**B**) P wave duration was significantly longer in the BO group (*n* = 9) than in the Control (*n* = 11) (***P < 0.001 vs. Control). PR interval and QRS duration were similar in the Control and the BO group (*P* = NS vs. Control by unpaired Student *t*-test). (**C**) Representative ECG recordings of Control (*upper panel*) and BO (*lower panel*) mice, respectively. Atrial fibrillation (AF) was induced by transesophageal atrial burst pacing (BP) at 10 min after norepinephrine injection (100 μg/kg, ip). The asterisk indicates P wave. SR: sinus rhythm. (**D**) The duration of AF was similar in the Control and BO groups at baseline, but was significantly increased by about twofold in the BO group (*n* = 3) at 10 min after NE injection (100 μg/kg, ip), compared to that in the Control (*n* = 3) (*P* < 0.05 vs. Control by unpaired student *t*-test). Data show means ± SD and scattered dots show individual data.

We examined the effects of BO on surface ECG extracted from the lead II (Fig. 2A) and found that the P wave duration was significantly longer in the BO group than in the control (Control (*n* = 9) vs. BO (n = 11): 9.7 ± 0.6 vs. 11.4 ± 1.1 ms, *P* < 0.001 vs. Control) (Fig. 2B). However, the PR interval and QRS duration was similar in the two groups (PR interval; Control (*n* = 9) vs. BO (n = 11): 7.9 ± 0.5 vs. 8.5 ± 1.1 ms, *P* = NS vs. Control; QRS duration; Control (*n* = 9) vs. BO (n = 11): 39.6 ± 2.9 vs. 39.1 ± 3.5 ms, *P* = NS vs. Control) (Fig. 2B).

Left atrial size was shown to be correlated with P wave duration on surface electrocardiogram (ECG)^29^ and its enlargement was shown to be an independent risk factor for AF in the Framingham study in humans^30,31^. More importantly, increased P wave duration was recently demonstrated to be a potent indicator of paroxysmal AF susceptibility and a discriminator of paroxysmal AF history not only in humans, but also in mice^32^. We thus examined the effects of BO on AF susceptibility in mice in terms of the duration of AF induced by transesophageal burst pacing^22,33–35^ (Fig. 2C).

The duration of AF was similar in the control and BO groups at baseline, but it was significantly increased in the BO group at 10 min after norepinephrine (NE) injection (100 μg/kg ip) (Control (*n* = 3) vs. BO (n = 3): 19.0 ± 6.6 vs. 41.0 ± 8.6 s, *P* < 0.05 vs. Control) (Fig. 2D).

These data suggest that AF susceptibility might be increased in the BO group, compared to that in the control mice, under conditions of the increased sympathetic activity.

### Effects of BO on fibrosis and myocyte apoptosis in atrium

**Figure 3:**
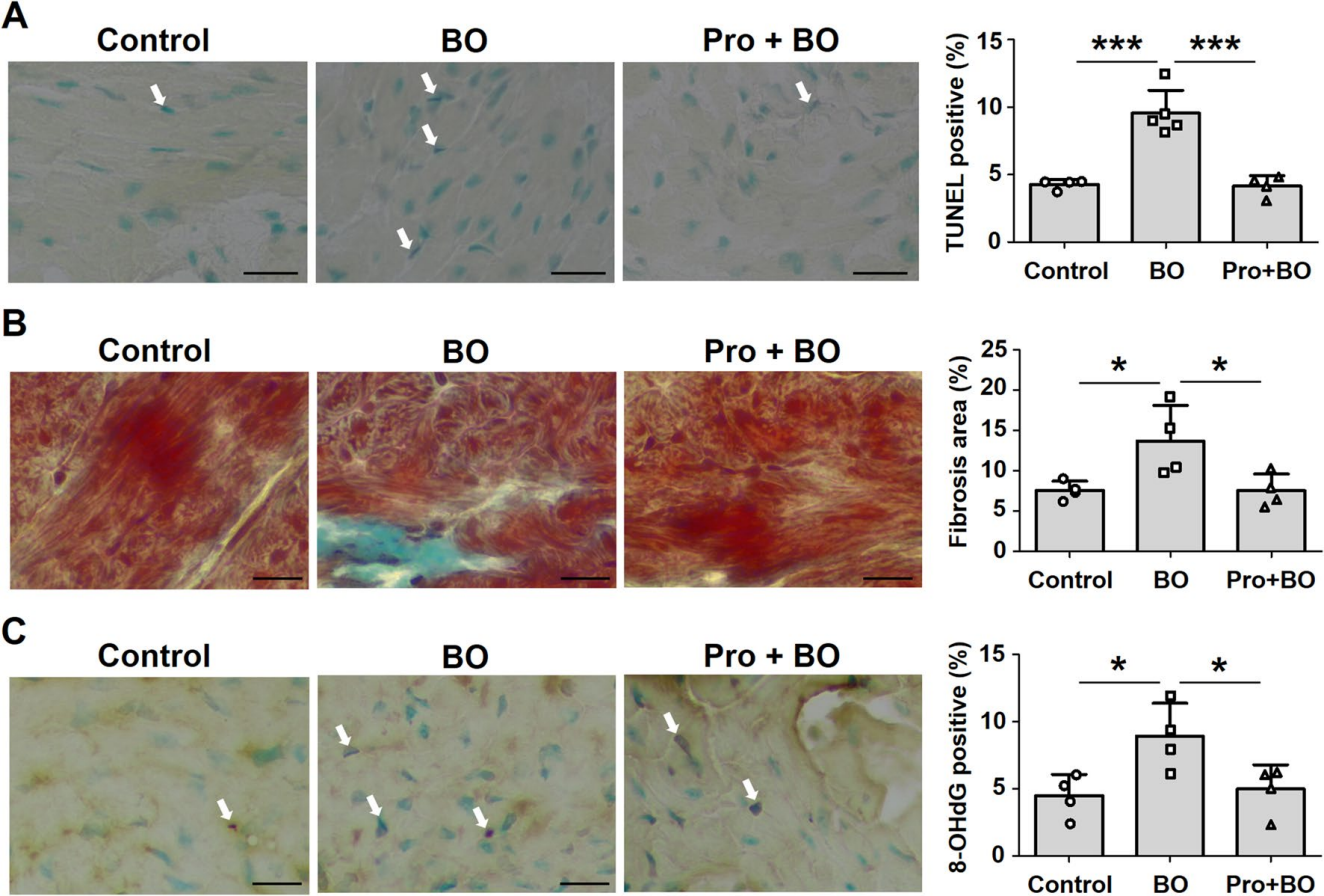
Effects of BO on fibrosis and myocyte apoptosis in atrium. (**A**) Representative images of TUNEL-stained sections of atrial muscle in the Control (*left*), BO (*middle*) and Pro + BO (*right*) groups. Scale bar: 20 μm. TUNEL-positive nuclei (white arrows) of atrial muscle were counted in the Control (*n* = 4), BO (*n* = 5) and Pro + BO (*n* = 4) groups at 2 weeks after BO treatment and expressed as percentage of total myocytes (*lower right*). The number of TUNEL-positive nuclei was significantly increased in the BO group (***P < 0.001 vs. Control by one-way ANOVA), but this increase was blocked in the Pro + BO group (***P < 0.001 vs. BO by one-way ANOVA). (**B**) Representative images of Masson-trichrome-stained sections of atrial muscle in the Control (*left*), BO (*middle*) and Pro + BO (*right*) groups. The area of fibrosis was significantly increased in the BO group (*n* = 4) (*P < 0.05 vs. Control (*n* = 4) by one-way ANOVA), but this increase was blocked in the Pro + BO group (*n* = 4) (***P < 0.001 vs. BO by one-way ANOVA). (**C**) Representative images of immunohistochemical detection of oxidative DNA damage (8-OHdG) of atrial muscle in the Control (*left*), BO (*middle*) and Pro + BO (*right*) groups. The number of 8OH-dG-positive nuclei was significantly increased in the BO group (*n* = 4) (*P < 0.05 vs. Control (*n* = 4) by one-way ANOVA), but this increase was blocked in the Pro + BO group (*n* = 4) (*P < 0.05 vs. BO by one-way ANOVA). Data show means ± SD and scattered dots show individual data.

Atrial structural remodeling is a prominent feature of AF and contributes to altered conduction^36,37^. We therefore examined atrial myocyte apoptosis by means of terminal deoxyribonucleotidyl transferase (TdT)-mediated biotin-16-deoxyuridine triphosphate (dUTP) nick-end labeling (TUNEL) staining (Fig. 3A) and fibrotic infiltration by means of Masson-trichrome staining (Fig. 3B).

TUNEL-positive cardiac myocytes were significantly increased by BO treatment (Control (*n* = 4) vs. BO (*n* = 5); 4.3 ± 0.4 vs. 9.6 ± 1.7%, *P* < 0.001 vs. Control), but this increase was blocked by co-treatment with propranolol (BO (*n* = 5) vs. Pro + BO (*n* = 4); 9.6 ± 1.7 vs. 4.2 ± 0.8%, *P* < 0.001 vs. BO) (Fig. 3A). The area of fibrosis in cardiac muscle was significantly increased in the BO group (Control (*n* = 4) vs. BO (*n* = 4); 7.6 ± 1.2 vs. 13.7 ± 4.4%, *P* < 0.05 vs. Control), but again propranolol blocked this increase (BO (*n* = 4) vs. BO + Pro (*n* = 4); 13.7 ± 4.4 vs. 7.5 ± 2.1%, *P* < 0.05 vs. BO) (Fig. 3B).

### Effects of BO on oxidative stress in atrium

Since oxidative stress plays an important role in the pathogenesis of AF^33,38^, we evaluated oxidative stress in the atria by 8-hydroxy-2′-deoxyguanosine (8-OHdG) immunostaining (Fig. 3C).

The ratio of 8-OHdG-positive/total cells was significantly increased by BO treatment (Control (*n* = 4) vs. BO (*n* = 4): 4.5 ± 1.6 vs. 8.9 ± 2.5%, *P* < 0.05 vs. Control), but propranolol blocked this increase (BO (*n* = 4) vs. Pro + BO (*n* = 4): 8.9 ± 2.5 vs. 5.0 ± 1.8%, *P* = 0.05 vs. BO) (Fig. 3C).

These results indicate that BO treatment increased oxidative stress-induced injury to atrial myocytes. This change, together with the increased levels of fibrosis and myocyte apoptosis in atrium, might contribute to the AF susceptibility of BO-treated mice.

### Effects of BO on CaMKII signaling in atrium

**Figure 4:**
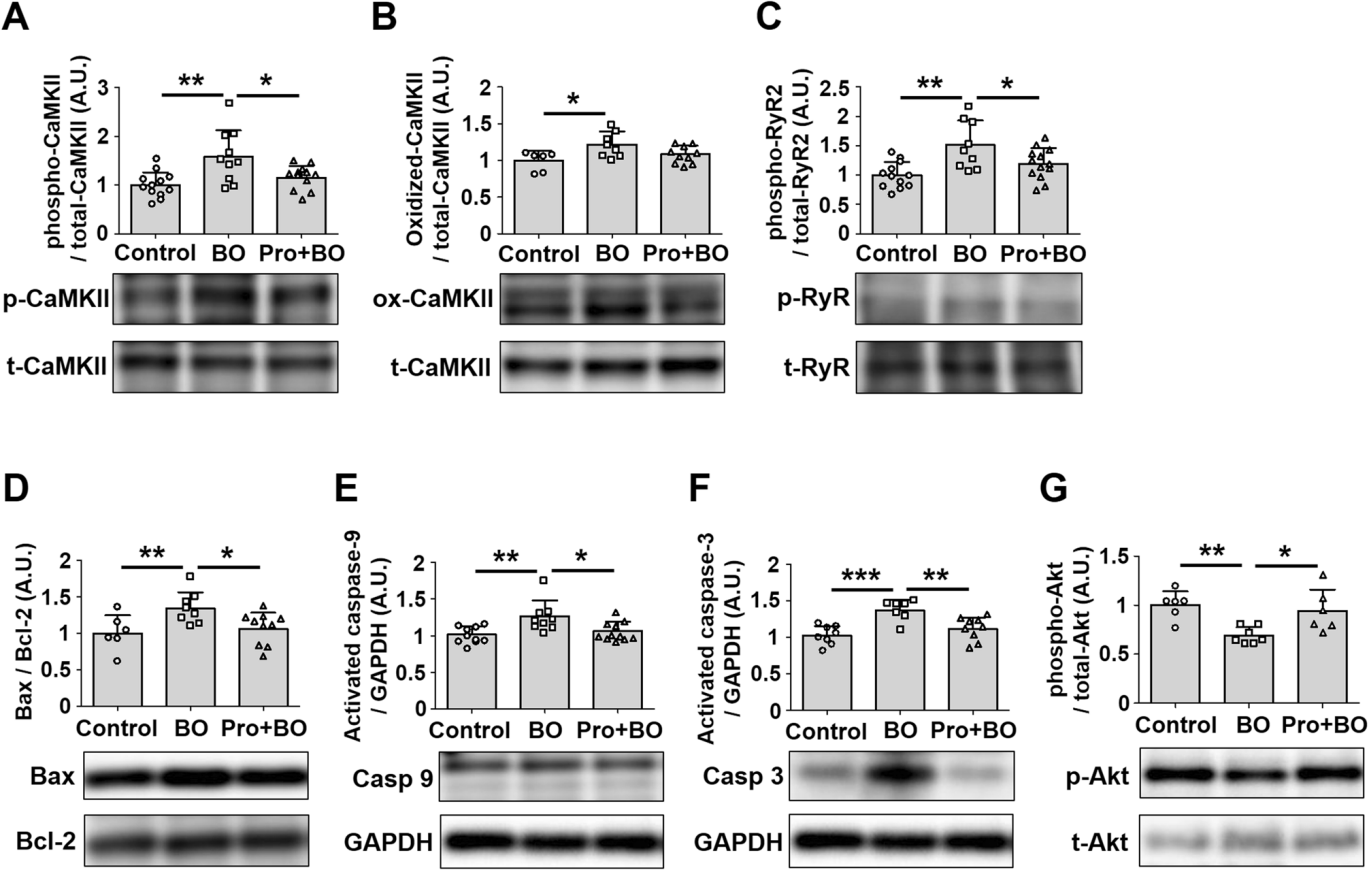
Effects of BO on CaMKII/RyR2 signaling, Bax/Bcl-2 ratio, activated caspase-3 expression and Akt phosphorylation in atrium. (**A**) CaMKII phosphorylation (Thr 286) was significantly increased in the BO group (*n* = 10) (**P < 0 .01 vs. Control (*n* = 12) by one-way ANOVA), but this increase was blocked in the Pro + BO group (*n* = 12) (**P < 0 .01 vs. BO by one-way ANOVA). Full-size images of immunoblots are presented in Supplementary Fig. 1. (**B**) CaMKII oxidation was significantly increased in the BO group (*n* = 8) (*P < 0 .05 vs. Control (*n* = 6) by one-way ANOVA). Full-size images of immunoblots are presented in Supplementary Fig. 2. (**C**) RyR2 phosphorylation (Ser-2814) was significantly increased in the BO group (*n* = 9) (**P < 0 .01 vs. Control (*n* = 12) by one-way ANOVA), but this increase was blocked in the Pro + BO group (*n* = 13) (*P < 0 .05 vs. BO by one-way ANOVA). Full-size images of immunoblots are presented in Supplementary Fig. 3. (**D**) The Bax/Bcl-2 ratio was significantly increased in the BO group (*n* = 8) (**P < 0 .01 vs. Control (*n* = 6) by one-way ANOVA), but this increase was blocked in the Pro + BO group (*n* = 10) (*P < 0 .05 vs. BO by one-way ANOVA). Full-size images of immunoblots are presented in Supplementary Fig. 4. (**E**) The level of activated caspase-9 was significantly increased in the BO group (*n* = 9) (**P < 0 .01 vs. Control (*n* = 10) by one-way ANOVA), but this increase was blocked in the Pro + BO group (*n* = 11) (*P < 0 .05 vs. BO by one-way ANOVA). Full-size images of immunoblots are presented in Supplementary Fig. 5. (**F**) The level of activated caspase-3 was significantly increased in the BO group (*n* = 7) (***P < 0 .001 vs. Control (*n* = 8) by one-way ANOVA), but this increase was blocked in the Pro + BO group (*n* = 9) (**P < 0 .01 vs. BO by one-way ANOVA). Full-size images of immunoblots are presented in Supplementary Fig. 6. (**G**) Akt phosphorylation (Ser-473) was significantly increased in the BO group (*n* = 7) (**P < 0 .01 vs. Control (*n* = 6) by one-way ANOVA), but this increase was blocked in the Pro + BO group (*n* = 6) (*P < 0 .05 vs. BO by one-way ANOVA). Full-size images of immunoblots are presented in Supplementary Fig. 7. Data show means ± SD and scattered dots show individual data.

Chronic activation of calmodulin-dependent protein kinase II (CaMKII) signaling results in cellular remodeling and alterations in calcium (Ca^2+^) handling, ion channels, cell-to-cell coupling and metabolism leading to increased risk of atrial fibrillation^37,39^. We thus examined the amounts of phospho-CaMKII (Thr-286) (Fig. 4A) and oxidized CaMKII (ox-CaMKII) (Fig. 4B) in the atrium of BO mice, and found that they were significantly increased (CaMKII (Thr-286): Control (*n* = 12) vs. BO (*n* = 10): 1.0 ± 0.3 vs. 1.6 ± 0.5 A.U., *P* < 0.01 vs. Control; ox-CaMKII: Control (*n* = 6) vs. BO (*n* = 8): 1.0 ± 0.1 vs. 1.2 ± 0.2 A.U., *P* < 0.05 vs. Control). The increase of CaMKII phosphorylation was blocked by propranolol (BO (*n* = 10) vs. Pro + BO (*n* = 12): 1.6 ± 0.5 vs. 1.2 ± 0.2 A.U., *P* < 0.05 vs. BO).

These data suggest that BO-induced CaMKII signaling activation might play a role, at least in part, in the development of AF susceptibility in the BO group via activation of β-AR signaling.

### Effects of BO on RyR2 phosphorylation on serine 2814 in atrium

CaMKII-mediated phosphorylation of ryanodine receptor 2 (RyR2) plays an important role in promoting AF susceptibility via RyR2-dependent Ca^2+^ leakage in mice^39^. We thus examined the expression levels of phospho-RyR2 (Ser-2814) in the atria of the three groups.

Phospho-RyR2 (Ser-2814) was significantly increased in the atrium of the BO group (Control (*n* = 12) vs. BO (*n* = 9): 1.0 ± 0.2 vs. 1.5 ± 0.4 A.U., *P* < 0.01 vs. Control), and this increase was blocked by propranolol (BO (*n* = 9) vs. Pro + BO (*n* = 13): 1.5 ± 0.4 vs. 1.2 ± 0.3 A.U. *P* < 0.05) (Fig. 4C).

These data suggest that BO-mediated AF susceptibility might be induced, at least in part, through the increase of RyR2 phosphorylation at Ser 2814.

### Effects of BO on Bax/Bcl-2 expression in atrium

We examined the effects of BO on the ratio of Bcl-2-associated protein (Bax), an accelerator of apoptosis, and B cell lymphoma-2 protein (Bcl-2), a regulator of apoptosis, in the atrium of the three groups (Fig. 4D) and found that it was significantly increased in the atrium of the BO group (Control (*n* = 6) vs. BO (*n* = 8); 1.0 ± 0.2 vs. 1.3 ± 0.2 A.U., *P* < 0.01 vs. Control). However, the increase was blocked by propranolol (BO (*n* = 8) vs. Pro + BO (*n* = 10); 1.3 ± 0.2 vs. 1.1 ± 0.2 A.U., *P* < 0.05 vs. Control), in accordance with the TUNEL staining data in Fig. 2A.

### Effects of BO on expression of cleaved-caspase-9 and caspase-3 in atrium

Caspase-9 mediates morphological change of mitochondria and ROS production. After activation of caspase-9, caspase-3 inhibits ROS production and is required for efficient execution of apoptosis^40^. We thus examined the expression levels of cleaved caspase-9 (Fig. 4E) and cleaved caspase -3 (Fig. 4F) in the three groups.

The expression of cleaved caspase-9 was significantly increased in the BO-group (Control (*n* = 10) vs. BO (*n* = 9); 1.0 ± 0.1 vs. 1.3 ± 0.2 A.U., *P* < 0.01 vs. Control), but propranolol blocked this increase (BO (*n* = 9) vs. Pro + BO (*n* = 11); 1.3 ± 0.2 vs. 1.1 ± 0.1 A.U., *P* < 0.05 vs. BO) (Fig. 4E).

The expression of cleaved-caspase-3 was also significantly increased in the BO group (Control (*n* = 8) vs. BO (*n* = 7); 1.0 ± 0.1 vs. 1.4 ± 0.1 A.U., *P* < 0.001 vs. Control), and again propranolol blocked the increase (BO (*n* = 7) vs. Pro + BO (*n* = 9); 1.4 ± 0.1 vs. 1.1 ± 0.2 A.U., *P* < 0.01 vs. Control) (Fig. 4F).

These data are consistent with the data of TUNEL results (Fig. 2A).

### Effects of BO on Akt phosphorylation

We then examined the effects of BO treatment on the level of Akt phosphorylation at serine 473 (Fig. 4G), since decreased Akt phosphorylation is involved in the pathogenesis of atrial remodeling and oxidative stress in the atrium, leading to AF^41,42^. Akt phosphorylation (Ser-473) was significantly decreased in the atrium of the BO group (Control (*n* = 6) vs. BO (n = 7): 1.0 ± 0.1 vs. 0.7 ± 0.1 A.U., *P* < 0.01 vs. Control). However, propranolol blocked the decrease of Akt phosphorylation (BO (*n* = 7) vs. Pro + BO (*n* = 6); 0.7 ± 0.1 vs. 0.9 ± 0.2 A.U., *P* < 0.05 vs. BO) (Fig. 4G).

These data suggest that BO-mediated atrial remodeling, oxidative DNA damage of atrial myocytes, and susceptibility to AF might be mediated, at least in part, through the decrease of cardioprotective Akt signaling^21,43^.

## Discussion

Mattila et al. first reported a significant association between oral disease and cardiovascular disease in 1989^6^. This link may be explained by chronic inflammation and repeated bacteremia from the oral cavity via periodontal disease, as inflammation plays an important role in the pathogenesis of atherosclerosis^44^. However, most studies showed a similar or stronger association between tooth loss and coronary heart disease, compared to periodontal disease and coronary heart disease, suggesting that periodontal disease may not completely explain the tooth loss-coronary heart disease relationship^12^.

Tooth loss may cause occlusal disharmony through the tipping of adjacent teeth toward the extraction area, extrusion of antagonist teeth, or unilateral mastification habits^45–47^. A recent study found that tooth loss showed a consistently strong and dose-dependent association with incidence of myocardial infarction, heart failure and stroke^48^. On the other hand, AF is one of the most prevalent arrhythmias, and increases the risk of stroke, heart failure, and overall mortality^1^. We thus hypothesized that occlusal disharmony might be a risk factor for AF. Our results here in BO mice represent the first evidence of a relationship between occlusal disharmony and atrial fibrillation.

We first examined the effect of BO on AF susceptibility in mice in terms of the duration of AF induced by transesophageal burst pacing^22,33^ (Fig. 2). We also demonstrated that the increased AF susceptibility of BO mice was mediated by increased CaMKII-mediated RyR2 phosphorylation at serine 2814, which might lead to increased spontaneous sarcoplastic reticulum Ca^2+^ leakage^39^. More importantly, co-treatment with a nonselective β-blocker, propranolol, abrogated the increased AF susceptibility in the BO group, suggesting that CaMKII-mediated RyR2 phosphorylation in the atrium of the BO group might be regulated by β-adrenergic signaling. We have recently demonstrated that the susceptibility to transesophageal rapid atrial pacing-induced AF and the levels of CaMKII-mediated phosphorylation on serine 2814 were decreased in mice lacking Epac1, a new target of cAMP signaling that is activated independently of PKA, and in mice treated with vidarabine, a cardiac AC inhibitor^22,34^. Further, type 5 AC, a major AC isoform in the heart, is preferentially coupled with β_1_-AR rather than β_2_-AR^49^. Our current and previous studies indicate that activation of β_1_-AR/type 5 AC/Epac1 signaling might be important for the BO-induced AF susceptibility.

**Figure 5:**
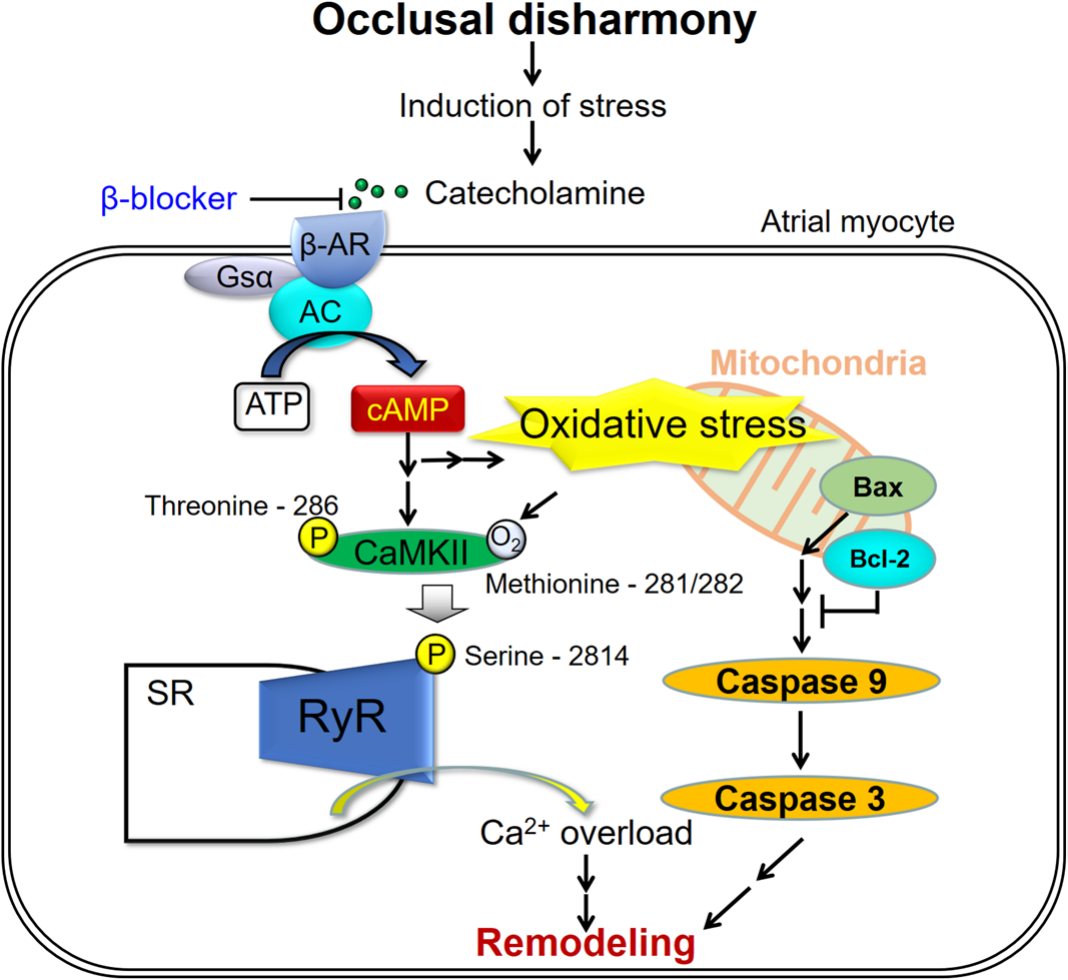
Schematic illustration of the proposed role of β-AR signaling in atrium. This scheme illustrates the proposed role of β-AR signaling in the atrium of BO-treated mice. BO-induced chronic stress causes prolonged β-AR activation, leading to the phosphorylation (Thr-286) of CaMKII, which mediates RyR2 phosphorylation (Ser-2814), leading to Ca^2+^ overload. In addition, the increased oxidative stress induces apoptotic signaling, such as an increase of Bax/Bcl-2 ratio, leading to activation of caspase-9 and caspase-3. These changes might cause fibrosis, myocyte apoptosis and oxidative stress in the atrium and AF susceptibility.

The duration of transesophageal burst pacing-induced AF was similar in the control and BO groups at baseline. However, it was significantly increased in the BO group compared with the control after catecholamine stimulation (100 μg/kg NE). These data suggest that, under conditions of increased CaMKII-mediated RyR2 phosphorylation and tissue abnormality in the atria, amplification of the altered RyR2 activation and subsequent diastolic SR Ca^2+^ leakage enhance the susceptibility to pacing-induced AF in the BO group (Fig. 5), as in the case of ventricular arrhythmia in mice with R176Q cardiac RyR2 mutations^50,51^.

Rhythmic chewing behavior has been shown to suppress endocrine stress response in the hypothalamus–pituitary–adrenal axis^52–54^, which was reported to be stimulated in BO-treated rats^13^.The hypothalamus plays an important role in central cardiovascular control^55^, and the demonstration of a negative correlation between chewing and activation of the hypothalamus–pituitary–adrenal axis suggests that natural occlusal force and perceived chewing ability could ameliorate stress-induced arrhythmia by inhibiting neuronal response in the hypothalamus, and the BO-induced AF susceptibility found in this study might be induced through the activation of the hypothalamus–pituitary–adrenal axis.

Poor oral health, particular amongst older people, is manifested in high levels of tooth loss and dental caries, and high prevalence of periodontal disease^56^. Also, extensive tooth loss reduces chewing performance and occlusal force, and has been linked to increase incidence of ischemic stroke, coronary heart disease and poor mental health in clinical studies, in addition to difficulty in eating property^48,56^. However, the mechanisms involved remain poorly understood.

To our knowledge, the present study is the first to examine the association between occlusal disharmony and AF, which is one of the most prevalent arrhythmias, and a risk factors for stroke, heart failure, and overall mortality. Our data suggest that improvement of occlusal disharmony by means of orthodontic treatment might be helpful in the treatment or prevention of AF.

## Limitations

It has been widely accepted that the induction of AF in mice is impossible due to the lack of a critical mass of the heart^57^. The murine atrial surface area is < 35 mm^2^, and therefore it may be difficult to sustain a micro-reentrant atrial tachyarrhythmia in an intact normal mouse. However, the AF mouse model induced by transesophageal atrial burst pacing can be used to evaluate mechanisms of arrhythmia using molecular and genetic approaches^22,33–35,58,59^. On the other hand, large animal models are needed for the macrostudy of atrial arrhythmia, and such studies have greatly contributed to our understanding of the underlying mechanisms. We plan to examine the effects of occlusal disharmony-induced stress on AF susceptibility using large animal models in the future.

In accordance with reported data^15,24^, BW was decreased after the BO treatment due to the acute decrease of food consumption following induction of BO, and then gradually increased nearly to the level of pre-BO treatment. However, weight loss was reported to improve cardiac function in mice with heart failure^60^, and we cannot exclude the possibility that weight loss might alter the phenotypes of the BO and Pro + BO groups shown in this study. We need to develop an occlusal disharmony mouse model with no change of body weight to confirm the findings of this study.

## Materials and methods

### Mice and experimental protocol

All experiments were performed on male 16-week-old C57BL/6 mice obtained from CLEA Japan (Tokyo, Japan). Occlusal disharmony in mice was induced by introducing a 0.7-mm BO, by cementing a suitable appliance onto the mandibular incisor under anesthesia with intraperitoneal medetomidine (0.03 mg/mL), midazolam (0.5 mg/mL), and butorphanol (0.5 mg/mL) ^15,28,61^ (Fig. 1A). Mice were group-housed at 23 °C under a 12–12 light/dark cycle with lights on at 8:00 AM and were divided into three groups: Control, a BO-only treatment group (BO) and a Pro + BO (Fig. 1B). ( ±)-Propranolol hydrochloride (#P0884, Sigma-Aldrich, St. Louis, MO, USA) was directly dissolved in drinking water (80 mg/kg/day; freshly prepared every day)^62^. Because BO mice cannot easily eat the standard pellet food (CE-2: 334.9 kcal/100 g) but can take paste food, the standard pellet food was changed to paste food 7 days before the BO treatment in all groups, as in previous studies^15,28^. Food intake, water intake and body weight of all animals were monitored throughout the 2-week experimental period. Animals were treated in accordance with institutional guidelines, and the experimental protocol was approved by the Animal Care and Use Committee of Tsurumi University. At the end of the physiological experiments, the animals were sacrificed by cervical dislocation and the hearts were quickly removed. The excised atria were weighed, then immediately frozen in liquid nitrogen with Tissue-Tek OCT compound (Sakura Finetek, Torrance, CA, USA), and stored at -80ºC until sectioning.

### Surface ECG analysis

Mice were anesthetized with isoflurane (1.0–1.5% vol/vol), and electrodes were attached to the limbs. Surface ECG extracted from lead II was obtained with a BioAmp (AD Instruments, Dunedin, New Zealand) and converted to digital form for analysis using LabChart (AD Instruments). Only high-quality ECGs were used in this study with high magnification (200–400%). PR intervals, QRS duration and P wave duration of three consecutive beats were measured by a single technician who was blind to information about the experimental groups, and mean values were used for analysis. All measurements were re-checked by an expert on cardiac physiology.

### Induction of AF

The induction of AF was performed as described previously by us^22,33,34^. Briefly, mice were anesthetized with isoflurane (1.5–2.0% vol/vol) to maintain adequate anesthesia, and norepinephrine bitartrate (Sigma-Aldrich) dissolved in natural saline (Otsuka Pharmaceutical, Tokyo, Japan) was intraperitoneally injected 10 min before AF induction. While a surface ECG was recorded using electrode in a lead-II configuration, a 1.1-French catheter electrode (EPR800; Millar Instruments, Houston, TX, USA) was carefully advanced and fixed at the esophageal position dorsal to the left atrium. Induction of AF was conducted by applying transesophageal atrial burst pacing for 10 s at a stimulation amplitude of 1.5 mA with 10 ms cycle lengths and a pulse width of 3 ms. AF was defined according to the following criteria: (i) loss of P wave, (ii) irregular R-R interval, and (iii) duration greater than 2 s, which have already been adopted for AF in mice induced by rapid transesophageal atrial pacing by us^33–35^ and other groups^58,59^. Further, after the screening of AF induction based on these criteria by a single technician who was blind to information about the experimental groups, the diagnosis was always re-checked by an expert on cardiac physiology.

### Evaluation of fibrosis and apoptosis

Cross sections (10 μm) were cut with a cryostat (CM1900, Leica Microsystems, Nussloch, Germany) at -20 ºC. The sections were air-dried and fixed with 4% paraformaldehyde (v/v) in 0.1 M phosphate-buffered saline (pH 7.5)^43,63,64^.

Interstitial fibrosis was evaluated by Masson-trichrome staining using the Accustain Trichrome Stain Kit (#HT15-1KT; Sigma-Aldrich) in accordance with the manufacturer’s protocol as described previously^22^. Interstitial fibrotic regions were quantified using Image J 1.48v software (National Institute of Health, Bethesda, MD, USA) to determine the percentage of blue-stained area in the Masson-trichrome sections^22,43,64^. DNA fragmentation was determined by TUNEL staining using an Apoptosis in situ Detection Kit (#293-71501; Wako, Osaka, Japan). The total number of TUNEL-positive nuclei was counted manually in six sections of three groups (Control (*n* = 4), BO (*n* = 5), and Pro + BP (*n* = 4)) over a microscopic field of 20 x, averaged, and expressed as the ratio of TUNEL-positive nuclei (%)^20,22,43,64^.

### Western blotting

Protein extraction and western blotting were performed based on previously described protocols^34,43,64^ with some modifications. The atrial tissue was ground in RIPA buffer (Thermo Fisher Scientific, Waltham, MA, USA) on ice. The homogenate was centrifuged at 15,000×*g* for 15 min at 4 °C. Protein concentration in the supernatant was determined by Bradford assay (Bio-Rad, Hercules, CA, USA). Samples containing equal amounts of protein were separated on 5–20% SDS–polyacrylamide gradient gel (Bio-Rad) and blotted onto polyvinylidene fluoride (PVDF) membrane (Bio-Rad). After treatment with blocking buffer (TOYOBO, Osaka, Japan) for 1 h at room temperature, the membrane was washed three times with Tris-buffered saline-0.05% (v/v) Tween20 (TBS-T). The membrane was incubated overnight at 4 °C with anti-phosphothreonine-286 CaMKII (1:1,000), anti-oxidized methionine-281/282 CaMKII (1:1,000) (Millipore, Billerica, MA, USA), anti-CaMKII (1:1,000) (CST), anti-phosphoserine-2814 RyR2 (1:5,000) (Badrilla, Leeds, UK), anti-RyR2 (1:1,000) (Thermo Fisher Scientific), anti-Bax (1:1,000), anti-Bcl-2 (1:1,000) (CST), anti-caspase-9 (1:500) (CST), anti-activated caspase-3 (1:200) (Abcam, Cambridge, UK), anti-glyceraldehyde-3-dehydrogenase (GAPDH) (1:200) (Santa Cruz Biotechnology, Santa Cruz, CA, USA), anti-phosphoserine-473 Akt (1:1,000) (CST), or anti-Akt (1:1,000) (CST) antibody. Membranes were washed with TBS-T, followed by incubation with horseradish peroxidase-conjugated secondary antibody (1:5,000) (GE Healthcare, Amersham, UK) for 1 h. Blots were visualized by chemiluminescence (GE Healthcare), and the density of signals was quantified using Image J software.

### Immunostaining

Oxidative DNA damage in the atrium was evaluated by immunostaining for 8-OHdG^65,66^. The sections were stained with anti-8-OHdG monoclonal antibody (clone N45.1, Japan Institute for the Control of Aging, Shizuoka, Japan) using the Vector M.O.M Immunodetection system (#PK-2200, Vector Laboratories, Inc. Burlingame, CA, USA). Briefly, after fixation with 4% (v/v) paraformaldehyde for 10 min at room temperature, the sections were incubated with N45.1 monoclonal antibody (7.5 μg/mL in M.O.M. Dilute) overnight at 4ºC in a humidified chamber, and then incubated in 0.3% H_2_O_2_ in 5% horse serum for 1 h to inactivate endogenous peroxidase, rinsed twice with TBS-T, incubated with biotinylated anti-mouse IgG in M.O.M. Diluent, and processed with an ABC kit (Vector Laboratories, Inc. Burlingame, CA, USA). We calculated the ratio of 8-OHdG nuclei with oxidative DNA damage, which stained dark blown, per total cell number.

### Statistics

All data are reported as mean ± standard deviations. Comparison of data was performed by Student’s *t* test for two groups, and one-way ANOVA followed by Tukey–Kramer’s post hoc test or two-way ANOVA followed by Bonferroni’s correction for 3 or more groups as indicated in each figure legend. Differences were considered significant at *P* < 0.05.

## Supplementary information

Supplementary file1

## Abbreviations

AF: Atrial fibrillation
BO: Bite-opening
β-AR: Beta-adrenergic receptor
AC: Adenylyl cyclase
ATP: Adenosine triphosphate
cAMP: Cyclic adenosine monophosphate
Epac: Exchange protein activated by cAMP
Control: Control group
Pro + BO: BO plus propranolol treatment group
NS: Not significant
BW: Body weight
ECG: Electrocardiogram
NE: Norepinephrine
TUNEL: Terminal deoxyribonucleotidyl transferase (TdT)-mediated biotin-16-deoxyuridine triphosphate (dUTP) nick-end labeling
8-OHdG: 8-Hydroxy-2′-deoxyguanosine
CaMKII: Calmodulin-dependent protein kinase II
Ca^2^^+^: Calcium
oxidized-CaMKII: Ox-CaMKII
RyR2: Ryanodine receptor 2
Bax: Bcl-2-associated protein
Bcl-2: B cell lymphoma-2 protein
PVDF: Polyvinylidene fluoride
TBS-T: Tris-buffered saline-0.05% (v/v) Tween20
GAPDH: Glyceraldehyde-3-dehydrogenase
