# Supplementary material for: Effects of occlusal disharmony on susceptibility to atrial fibrillation in mice
Source: Sci Rep. 2020 Aug 13;10:13765. doi: 10.1038/s41598-020-70791-8 (PMC7426945; doi:10.1038/s41598-020-70791-8)
Supplement: Supplementary file 1 — Supplementary file1 [file 41598_2020_70791_MOESM1_ESM.pdf]

Effects of occlusal disharmony on susceptibility to atrial fibrillation in mice

Kenji Suita [a], Yuka Yagisawa [b], Yoshiki Ohnuki [a], Daisuke Umeki [b], Megumi Nariyama [c], Aiko Ito [b], Yoshio Hayakawa [e], Ichiro Matsuo [d], Yasumasa Mototani [a], Yasutake Saeki [a], Satoshi Okumura [a, *]

[a] Department of Physiology; [b] Department of Orthodontics; [c] Department of Pediatric dentistry; [d] Department of Periodontology; [e] Department of Anesthesiology, Tsurumi University School of Dental Medicine, Yokohama 230-8501, Japan.

Supplementary Figures

Supplementary Figure 1

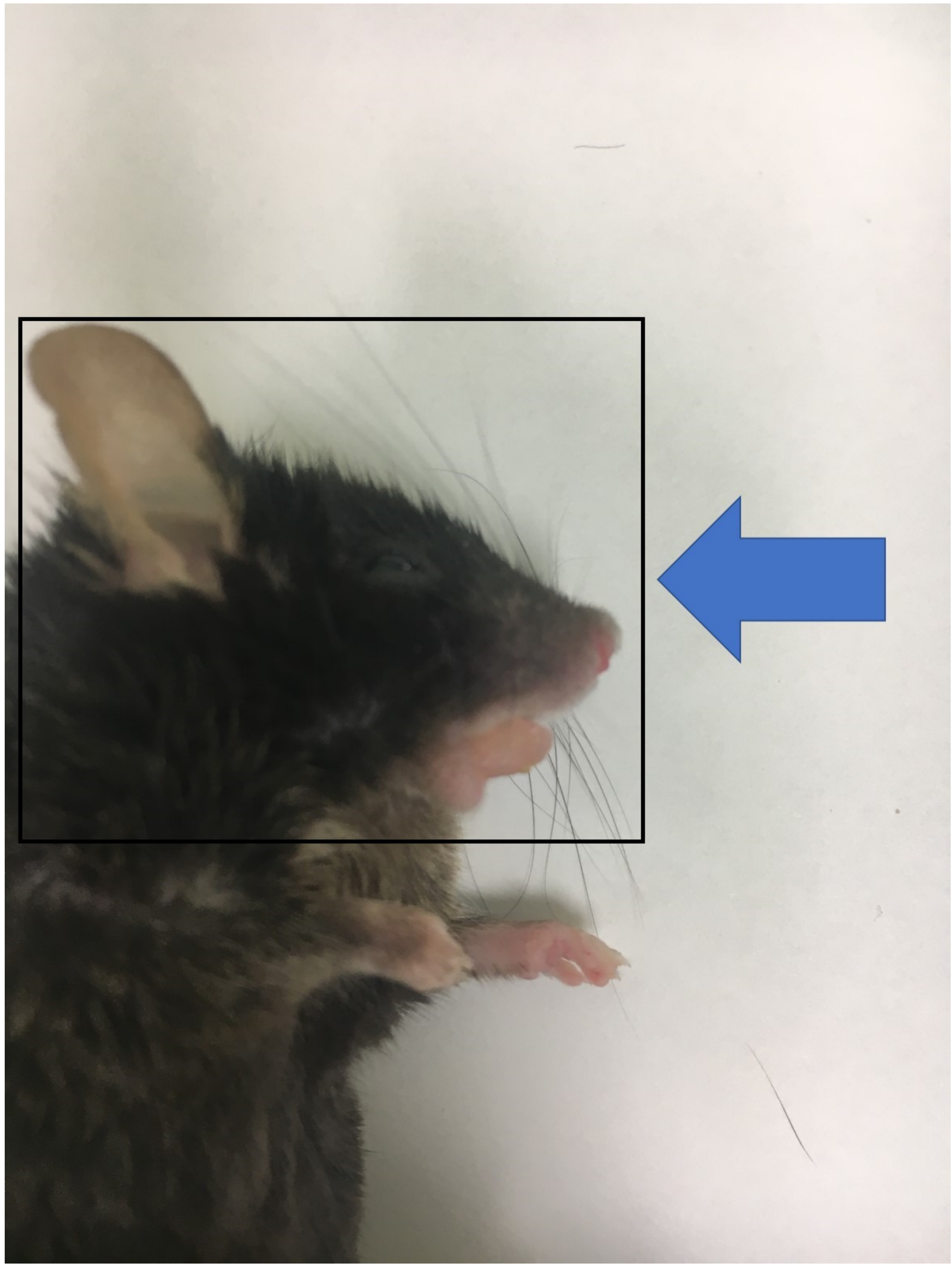

Supplementary Figure 1. The original photo of Fig. 1A. The black-line box indicated by the arrow corresponds to the cropped part that is shown in the main article.

Supplementary Figure 2

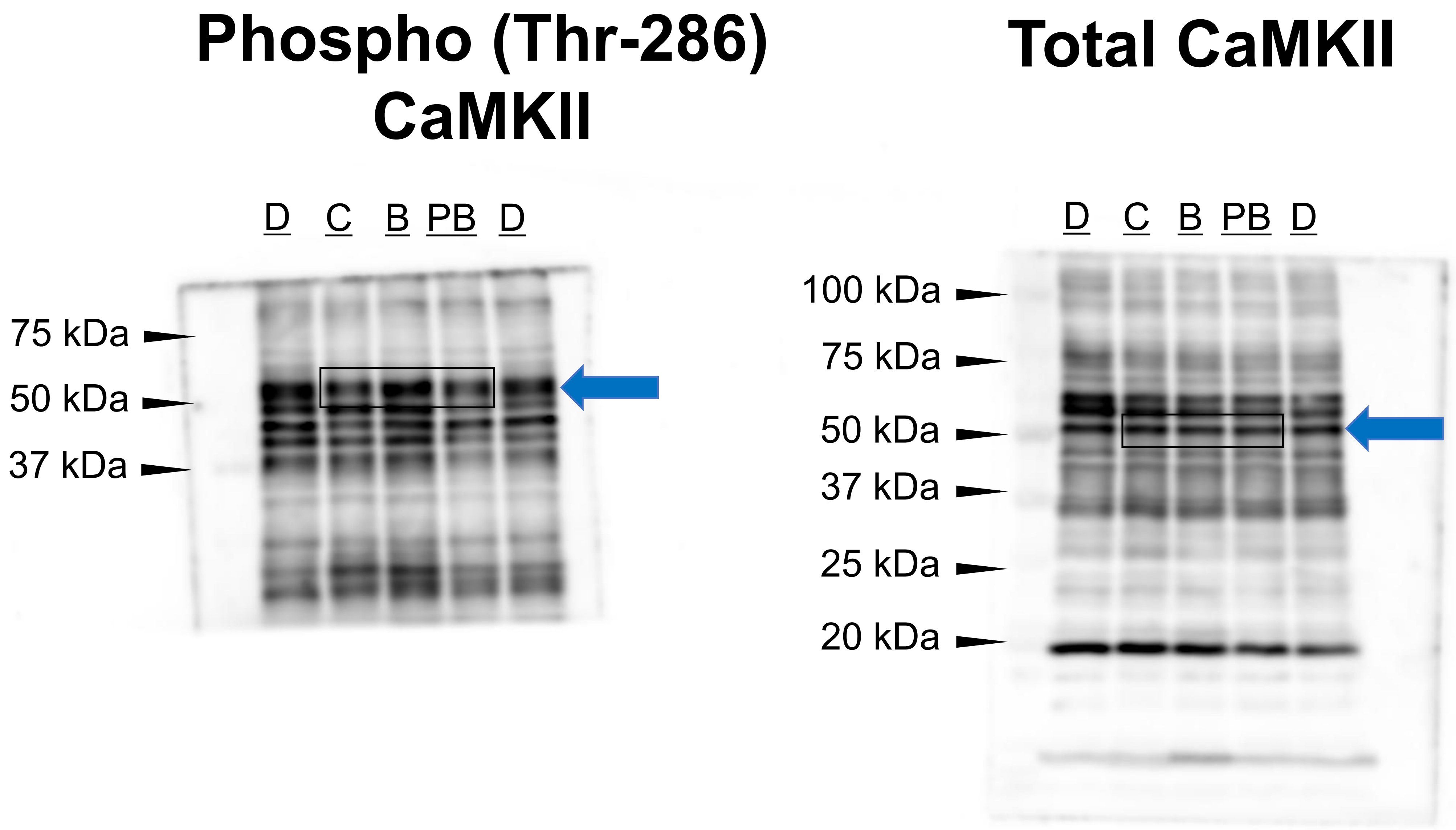

Supplementary Figure 2. Representative full-length immunoblots of Fig. 4C. The amounts of phosphorylated CaMKII at Thr-286 (left panel) and total CaMKII (right panel) are shown. The black-line box indicated by the arrow in each blot corresponds to the cropped parts that are shown in the main article. C: control, B: BO, PB: Propranolol + BO, D: dummy sample.

Supplementary Figure 3

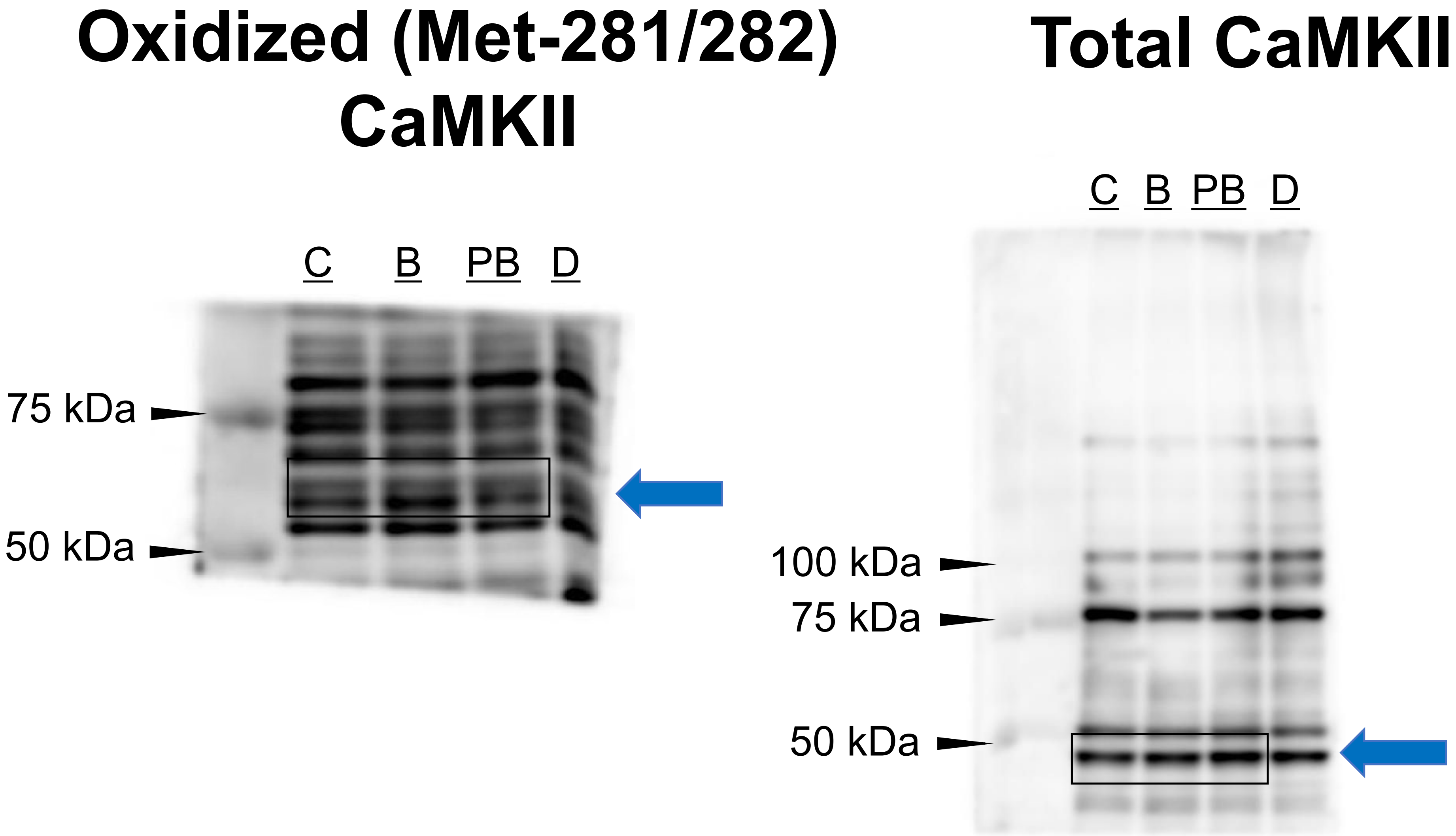

Supplementary Figure 3. Representative full-length immunoblots of Fig. 4C. The amounts of oxidized CaMKII at Met-281/282 (left panel) and total CaMKII (right panel) are shown. The black-line box indicated by the arrow in each blot corresponds to the cropped parts that are shown in the main article. C: control, B: BO, PB: Propranolol + BO, D: dummy sample.

Supplementary Figure 4

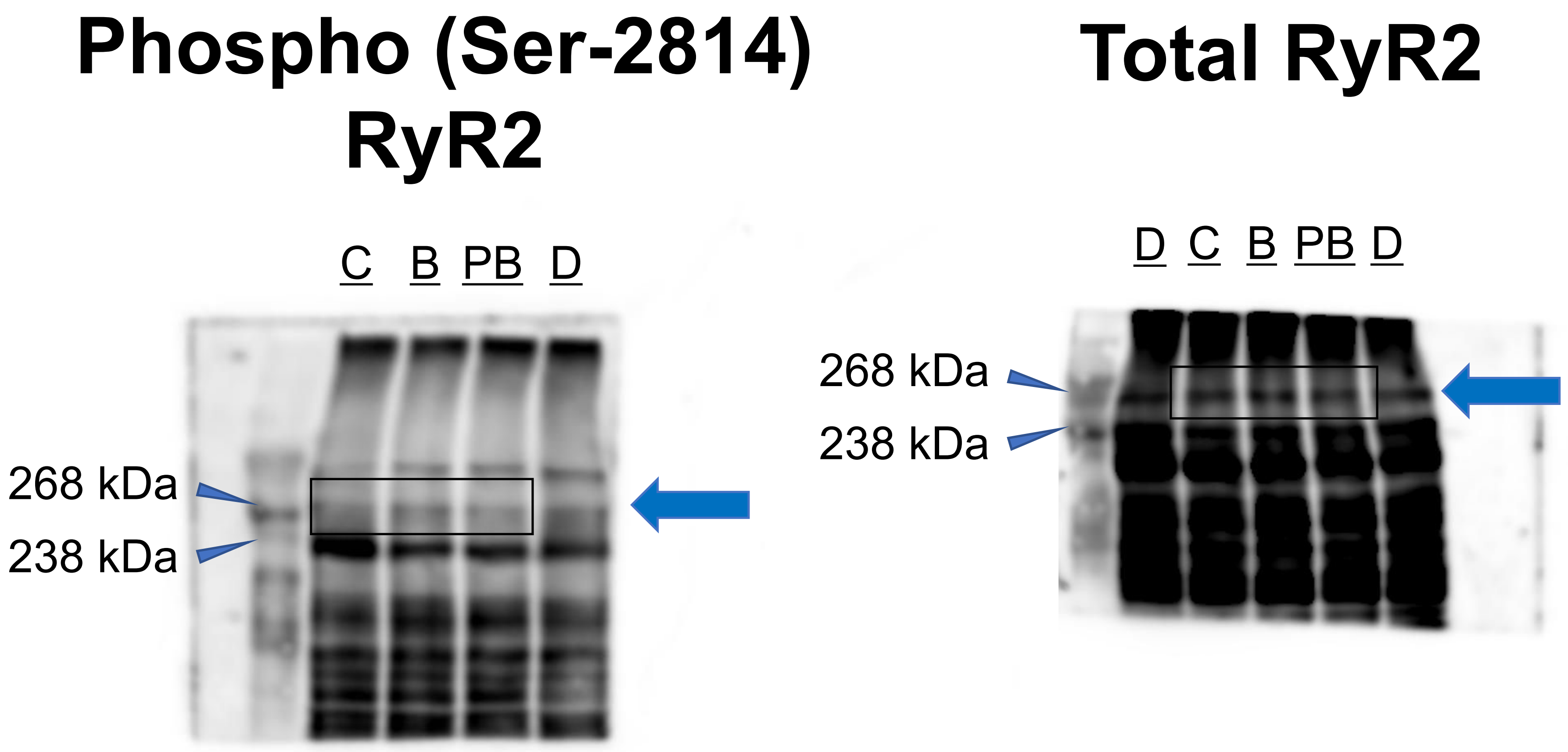

Supplementary Figure 4. Representative full-length immunoblots of Fig. 4C. The amounts of phosphorylated RyR2 at Ser-2814 (left panel) and total RyR2 (right panel) are shown. The black-line box indicated by the arrow in each blot corresponds to the cropped parts that are shown in the main article. C: control, B: BO, PB: Propranolol + BO, D: dummy sample.

Supplementary Figure 5

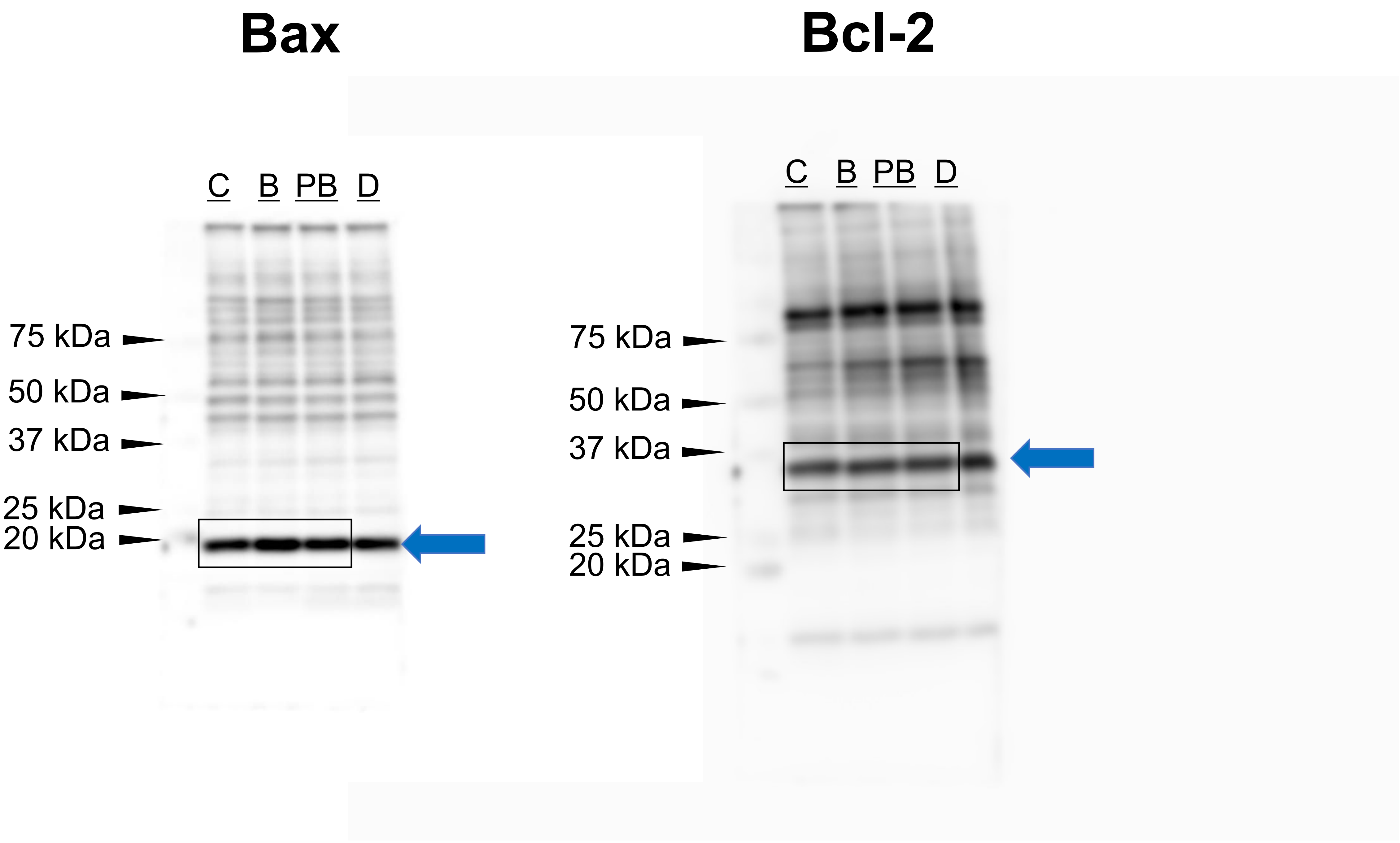

Supplementary Figure 5. Representative full-length immunoblots of Fig. 4D. The amounts of Bax (left panel) and total Bcl-2 (right panel) are shown. The black-line box indicated by the arrow in each blot corresponds to the cropped parts that are shown in the main article. C: control, B: BO, PB: Propranolol + BO, D: dummy sample.

Supplementary Figure 6

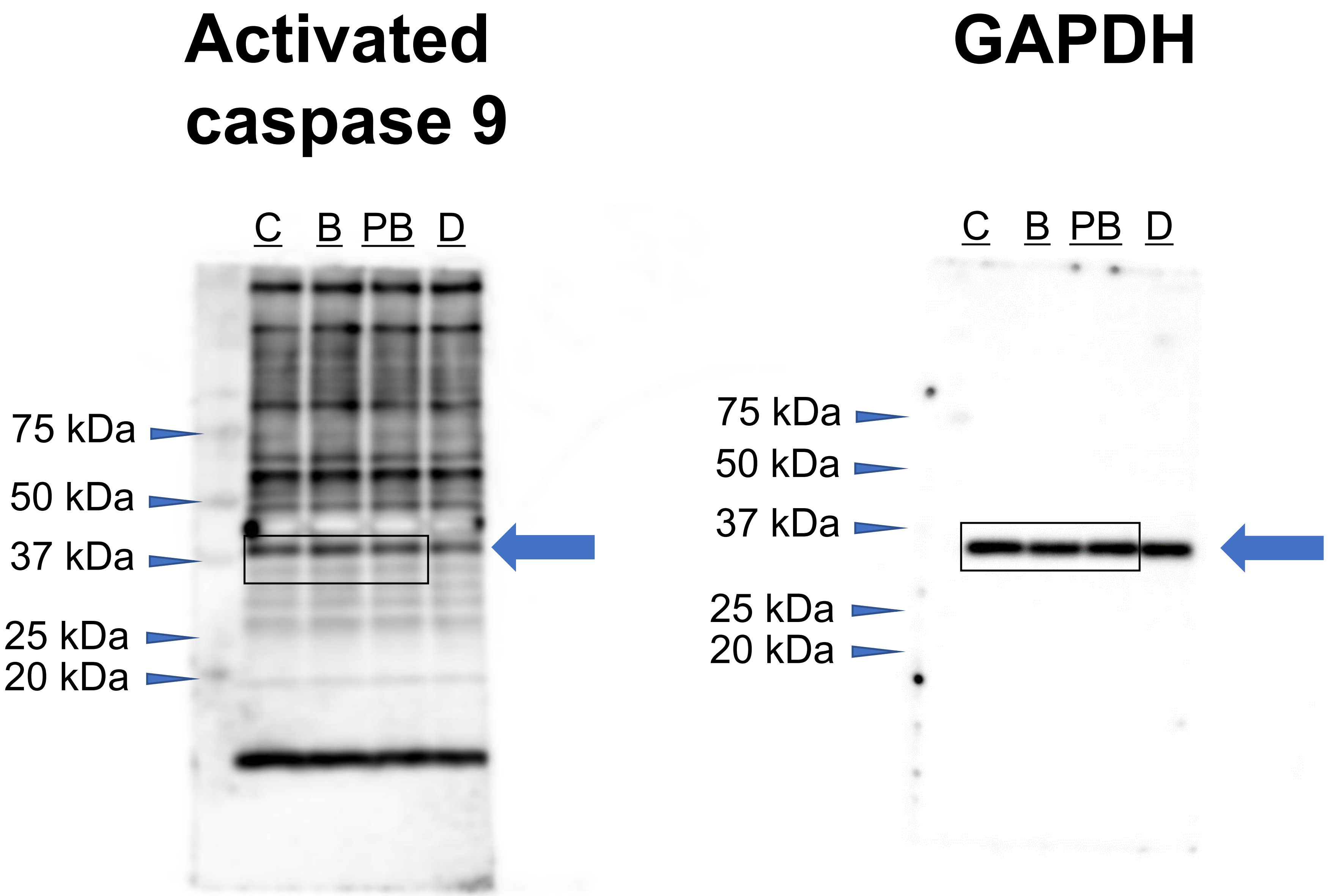

Supplementary Figure 6. Representative full-length immunoblots of Fig. 4E. The amounts of activated caspase 9 (left panel) and GAPDH (right panel) are shown. The black-line box indicated by the arrow in each blot corresponds to the cropped parts that are shown in the main article. C: control, B: BO, PB: Propranolol + BO, D: dummy sample.

Supplementary Figure 7

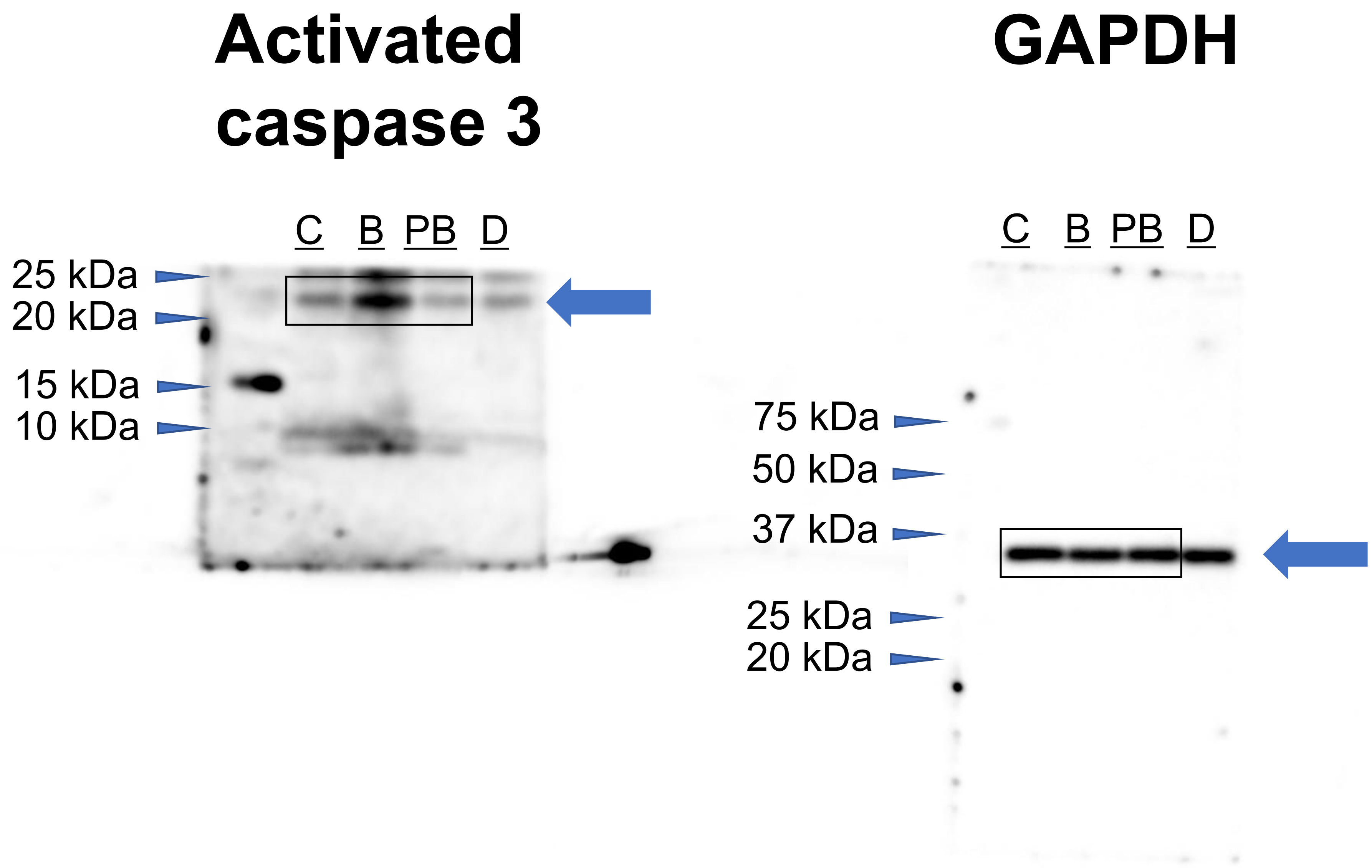

Supplementary Figure 7. Representative full-length immunoblots of Fig. 4F. The amounts of activated caspase 3 (left panel) and GAPDH (right panel) are shown. The black-line box indicated by the arrow in each blot corresponds to the cropped parts that are shown in the main article. C: control, B: BO, PB: Propranolol + BO, D: dummy sample.

Supplementary Figure 8

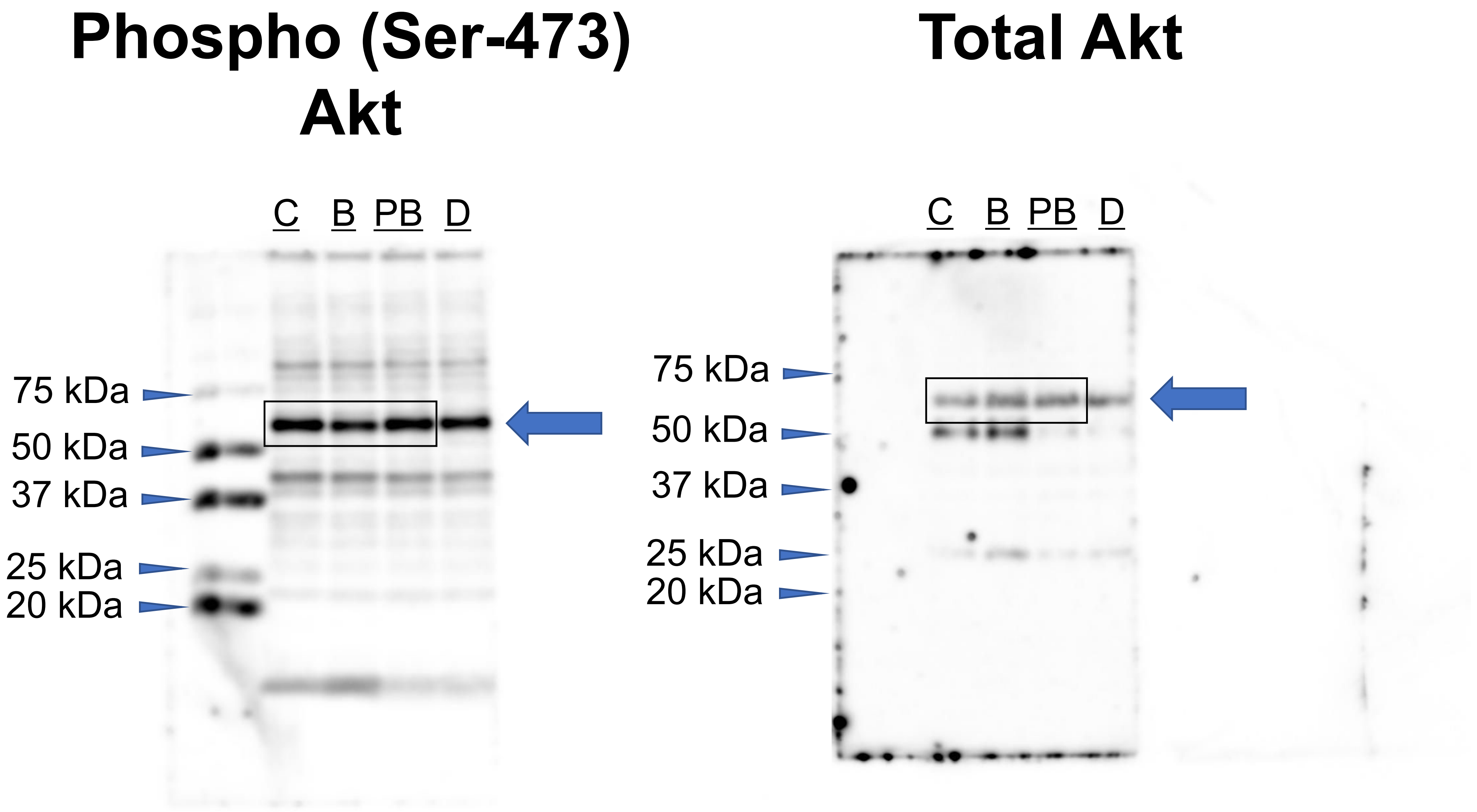

Supplementary Figure 8. Representative full-length immunoblots of Fig. 4G. The amounts of phosphorylated Akt at Ser-473 (left panel) and total Akt (right panel) are shown. The black-line box indicated by the arrow in each blot corresponds to the cropped parts that are shown in the main article. C: control, B: BO, PB: Propranolol + BO, D: dummy sample.
